# Ejection Fraction-Related Differences of Baseline Characteristics and Outcomes in Troponin-Positive Patients without Obstructive Coronary Artery Disease

**DOI:** 10.3390/jcm13102826

**Published:** 2024-05-11

**Authors:** Mustafa Kacmaz, Clara Schlettert, Fabienne Kreimer, Mohammad Abumayyaleh, Ibrahim Akin, Andreas Mügge, Assem Aweimer, Nazha Hamdani, Ibrahim El-Battrawy

**Affiliations:** 1 Institute of Physiology, Department of Cellular and Translational Physiology and Institute für Forschung und Lehre (IFL), Molecular and Experimental Cardiology, Ruhr-University Bochum, 44791 Bochum, Germany; mustafa.kacmaz@ruhr-uni-bochum.de (M.K.); assem.aweimer@bergmannsheil.de (A.A.); nazha.hamdani@ruhr-uni-bochum.de (N.H.); 2 HCEMM-SU Cardiovascular Comorbidities Research Group, Department of Pharmacology and Pharmacotherapy, Semmelweis University, 1089 Budapest, Hungary; 3 Department of Cardiology and Angiology, Bergmannsheil University Hospital, Ruhr University of Bochum, 44789 Bochum, Germany; cschlettert@gmx.de; 4 Department of Cardiology and Rhythmology, University Hospital St. Josef Hospital Bochum, Ruhr University Bochum, 44791 Bochum, Germany; fabienne.kreimer@t-online.de (F.K.); andreas.muegge@bergmannsheil.de (A.M.); 5 First Department of Medicine, University Medical Centre Mannheim (UMM), 68167 Mannheim, Germany; mohammad.abumayyaleh@umm.de (M.A.); ibrahim.akin@umm.de (I.A.); 6 Department of Physiology, Cardiovascular Research Institute Maastricht, University Maastricht, 6200 Maastricht, The Netherlands

**Keywords:** troponin elevation, in-hospital complications, ejection fraction, myocardial infarction, coronary artery disease

## Abstract

**Background:** The development and course of myocardial infarction with non-obstructive coronary artery (MINOCA) disease is still not fully understood. In this study, we aimed to examine the baseline characteristics of in-hospital outcomes and long-term outcomes of a cohort of troponin-positive patients without obstructive coronary artery disease based on different left ventricular ejection fractions (LVEFs). **Methods and results:** We included a cohort of 254 patients (mean age: 64 (50.8–75.3) years, 120 females) with suspected myocardial infarction and no obstructive coronary artery disease (MINOCA) in our institutional database between 2010 and 2021. Among these patients, 170 had LVEF ≥ 50% (84 females, 49.4%), 31 patients had LVEF 40–49% (15 females, 48.4%), and 53 patients had LVEF < 40% (20 females, 37.7%). The mean age in the LVEF ≥ 50% group was 61.5 (48–73) years, in the LVEF 40–49% group was 67 (57–78) years, and in the LVEF < 40% group was 68 (56–75.5) years (*p* = 0.05). The mean troponin value was highest in the LVEF < 40% group, at 3.8 (1.7–4.6) µg/L, and lowest in the LVEF ≥ 50% group, at 1.1 (0.5–2.1) µg/L (*p* = 0.05). Creatine Phosphokinase (CK) levels were highest in the LVEF ≥ 50% group (156 (89.5–256)) and lowest in the LVEF 40–49% group (127 (73–256)) (*p* < 0.05), while the mean BNP value was lowest in the LVEF ≥ 50% group (98 (48–278) pg/mL) and highest in the <40% group (793 (238.3–2247.5) pg/mL) (*p* = 0.001). Adverse in-hospital cardiovascular events were highest in the LVEF < 40% group compared to the LVEF 40–49% group and the LVEF ≥ 50% group (56% vs. 55% vs. 27%; *p* < 0.001). Over a follow-up period of 6.2 ± 3.1 years, the all-cause mortality was higher in the LVEF < 40% group compared to the LVEF 40–49% group and the LVEF ≥ 50% group. Among the different factors, LVEF < 40% and LVEF 40–49% were associated with an increased risk of in-hospital cardiovascular events in the multivariable Cox regression analysis. **Conclusions**: LVEF has different impacts on in-hospital cardiovascular events in this cohort. Furthermore, LVEF influences long-term all-cause mortality.

## 1. Introduction

Acute myocardial infarction (AMI) is associated with a high mortality ratio of up to 42% [1,2,3]. In most cases of acute myocardial ischemia cases, the reason is defined as coronary heart disease with significant stenosis or occlusion of one or more main coronary arteries. In about 10% of these patients with acute myocardial infarction, significant stenosis cannot be diagnosed. This phenomenon is known as myocardial infarction with non-obstructive coronary artery (MINOCA) [4,5]. MINOCA is one of most common causes of cardiac death [6]. Although this phenomenon was documented a couple of years ago, the heterogeneous clinical profile of the disease and its pathophysiological mechanism remain elusive, therefore making it difficult to classify and develop effective treatments [5,6]. According to the fourth universal definition of myocardial infarction, MINOCA should be diagnosed in cases where there is evidence of an AMI that occurs spontaneously and no coronary stenosis of 50% or more in the main epicardial vessels [7].

In suspected MINOCA cases, cardiac magnetic resonance (CMR) imaging could assist in confirming the diagnosis. In more than half of the suspected MINOCA cases, CMR imaging can exclude MINOCA as a definitive diagnosis. This underscores the significance of CMR for diagnostic and prognostic reasons]. In addition, CMR diagnoses of acute myocardial infarction, myocarditis, and nonischemic cardiomyopathy are independent predictors of poor outcomes [8].

The main mechanisms of MINOCA are mainly related to myocardial ischemia triggered by various causes (such as coronary spontaneous dissection, epicardial coronary vasospasm, coronary thrombi/embolism). In addition, age, sex, arterial hypertension, and dyslipidemia may trigger the development of MINOCA [9,10]. In the absence of significant obstruction of the coronary arteries, increased serum troponin as a specific marker of myocardial necrosis is present in MINOCA [10,11,12].

Heart failure is a common and important cardiovascular risk factor event in the MINOCA cohort [13]. A registry-based TOTAL-AMI study examined the outcome of MINOCA patients at a median follow-up of 3.4 years. They found that MINOCA patients had a high prevalence of heart failure and 27.6% of patients had impaired LVEF [14]. Of note, LVEF has different impacts on in-hospital complications and cardiovascular events over follow-up in myocardial infarction with obstructive coronary artery disease [15,16,17]. However, the impact of LVEF variation on the outcome of MINOCA has not been well studied yet.

In the present study, patients with suspected MINOCA were investigated. Baseline characteristics, in-hospital complications, and long-term adverse events related to several ejection fraction profiles in MINOCA and suspected MINOCA patients were studied. 

## 2. Method

**Figure 1:**
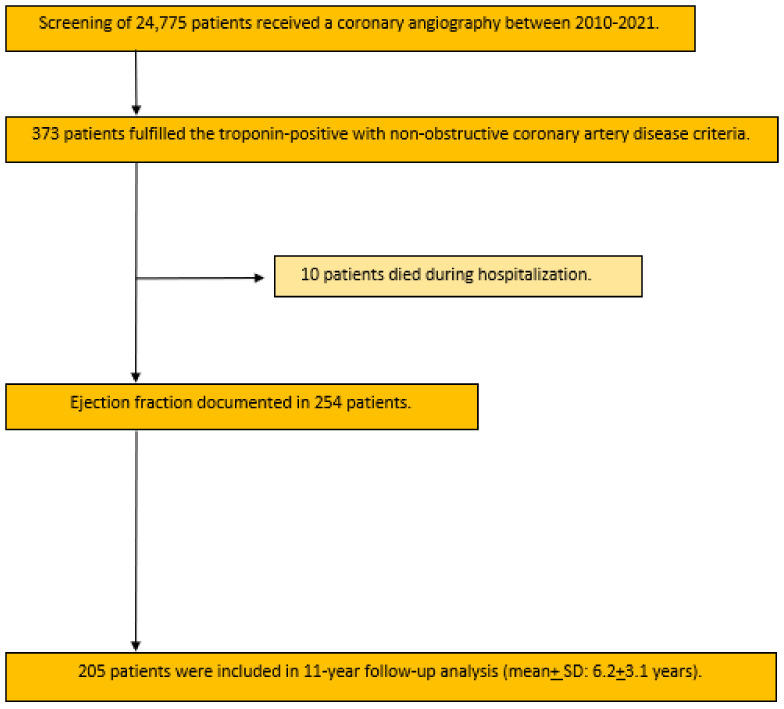
Flow-chart presenting the screened data and included patients for the present study.

**Figure 2:**
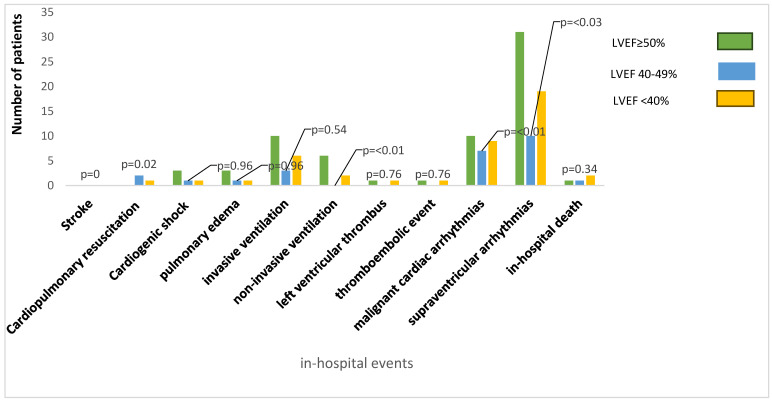
Intra-hospital events related to ejection fraction.

The schematic template of the study process and intra-hospital events related to LVEF are shown in Figure 1 and Figure 2. In the current study, a monocentric retrospective analysis of clinical data was conducted based on a total of 24,775 patients who underwent coronary angiography at Bergmannsheil University Hospital between January 2010 and April 2021.

### 2.1. Study Population

Of these cases, 254 patients fulfilled the required inclusion criteria and information about LVEF was available. Among them, 170 patients had LVEF ≥ 50%, 31 patients had LVEF 40–49%, and 53 patients had LVEF < 40%. A total of 10 patients died during hospitalization; 205 patients were included in the follow-up analysis. The follow-up time was 6.2 + 3.1 years (Figure 1). 

### 2.2. Diagnosis

LVEF values were measured with the assistance of a biplane echocardiography according to ASE/EACVI guidelines [18,19]. The echocardiography at admission was performed within 1 day after admission and the LVEF measurement before discharge was performed within 1–2 days before discharge. Patients initially fulfilled the modified criteria for AMI [20]. Troponin levels were measured using the high-sensitivity Troponin I test. Afterwards, proof of an infarction was demonstrated, which could be shown by at least one of the following criteria: myocardial ischemia symptoms, fresh ischemic changes on the electrocardiogram, pathologic Q waves, signs of fresh loss of viable myocardium or fresh regional wall motion, abnormalities consistent with an ischemic cause, or proof of a coronary thrombus through angiography or autopsy. Additionally, the exclusion of coronary artery obstruction was needed for patients who received angiography to exclude a stenosis of more than 50%. Moreover, nonspecific alternative diagnoses that might have caused the clinical presentation needed to be excluded from the scope of the study. Participants under the age of 18 and those whose datasets missed critical information were excluded from the study.

Expert cardiologists independently determined the diagnosis based on evaluations of angiograms, echocardiograms, and laboratory results. However, only 71 of the patients underwent CMR. We collected demographic data and cardiovascular risk variables from the present medical records. Results of the coronary angiography and details on the medication prescribed for discharge were recorded throughout the hospitalization period. The study protocol obtained approval by the Medical Faculty Ethics Committee at Ruhr University Bochum, approval number 22-7684, on the 16 October 2022.

Stroke, cardiopulmonary resuscitation, cardiogenic shock, pulmonary edema, invasive and non-invasive ventilation, left ventricular thrombus, thromboembolic events, life-threatening cardiac arrhythmias, supraventricular arrhythmias, and all-cause mortality were all included in the study’s primary endpoint of adverse events. Medical data were tackled to determine each of these incidents. During the follow-up period of 6.2 + 3.1 years, extra-hospital events such as stroke, thromboembolic events, adverse events, recurrence of elevated troponin positive value and subsequent re-admission to the hospital, percutaneous coronary intervention (PCI), cardiac arrest, and all-cause mortality were assessed. 

To assist the follow-up, treating physicians were contacted. In case no information was available, patients were notified over the phone. In case of an unknown cause of death, this was stated as unknown cause. Patients who were not reachable by phone were contacted by relatives to complete the follow-up as far as possible.

### 2.3. Statistical Analysis

Mean ± standard deviation is used to represent continuous variables with a normal distribution, while median (interquartile range) is used to represent continuous variables with a non-normal distribution. Categorical variables are expressed as counts and relative frequencies (%). To evaluate normality, the Shapiro–Wilk test was employed. The Mann–Whitney U test and Student’s *t*-test for independent samples were used to compare continuous variables with normal and non-normal distributions, respectively. Categorical variables were compared using the Chi-square test and Fisher’s exact test. The statistical analysis was performed using SPSS Statistics 28.0 software, with a significance threshold of *p* ≤ 0.05. A multivariate Cox regression model that was adjusted for confounding factors included factors with a *p*-value of less than 0.05 in univariate analysis to address potential baseline characteristics differences that might affect outcomes.

## 3. Results

### 3.1. Baseline Characteristics

Figure 1 presents a flow-chart of the study cohort. 

The male-to-female ratio was nearly 1:1 (52.7% male) and the median age was 64 (50.8–75.3) years. On admission, the median LVEF values were 60 (55–65) % vs. 45 (40–46) % and 30 (23–35) % among groups; *p* < 0.001. Before discharge, the LVEF values in all groups were 60 (56–65) % vs. 40.5 (34.8–50) % and 38 (27–50); *p* < 0.001 respectively (Table S1). The median age was not significantly different between the groups (LVEF ≥ 50% compared to LVEF 40–49% and EF < 40%; *p* = 0.05). Angina pectoris was the main symptom in LVEF ≥ 50% (67.7%) compared to LVEF 40–49% (54.8%) and LVEF < 40% (32.1%); *p* < 0.001. Nevertheless, patients with LVEF < 40% were more complaining about dyspnea (EF < 40% 71.7% vs. LVEF 40–49% 51.6% and LVEF ≥ 50% 33.7%; *p* < 0.001). Dyslipidemia was numerically more common in LVEF ≥ 50% (29.4%) compared to LVEF 40–49% (16.1%) and LVEF < 40% (16.9%); *p* = 0.05. In the medical history among comorbidities, malignancy and kidney disease were significantly higher documented in LVEF < 40% compared to LVEF 40–49% and EF ≥ 50%; *p* = 0.04 and *p* = 0.006. On admission, laboratory results showed increased levels of the following cardiac biomarkers: elevated troponin value in LVEF < 40% 3.8 (1.7–4.6) µg/L compared to LVEF 40–49% 1.3 (0.8–2.3) µg/L, and EF ≥ 50 1.1 (0.5–2.1) µg/L; *p* = 0.06. CK levels were highest in LVEF ≥ 50% 156 (89.5–256) U/L compared to LVEF 40–49% 127 (73–256) U/L and LVEF < 40% 133.5 (68–305.5) U/L; *p* < 0.05. BNP levels were highest in LVEF < 40% 793 (238.3–2247.5) pg/mL compared to LVEF 40–49% 266.5 (162.5–602.3) pg/mL, and LVEF ≥ 50% 98 (48–278) pg/mL; *p* < 0.001 (Table 1). 

Except diuretics no statistically significance observed among “medication at discharge” groups (Table 2).

### 3.2. In-Hospital Complications

Duration of hospitalization was shorter in LVEF ≥ 50% 7 (6–9) days compared to LVEF 40–49% 8 (6–16) days and LVEF < 40% 12 (8–17) days; *p* < 0.001, which could be explained by the different in-hospital adverse event rate. Adverse in-hospital events were lowest in LVEF ≥ 50% (27.1%) compared to LVEF 40–49% (54.8%) and LVEF < 40% (55.8%); *p* < 0.001. Although in-hospital complications, e.g., left ventricular thrombus, thromboembolic events, pulmonary edema, cardiogenic shock, invasive ventilation, and stroke, presented similarly in all groups, LVEF 40–49% suffered more significantly from malignant arrhythmic events. The in-hospital all-cause mortality did not differ significantly between the groups (Table 3, Figure 2).

### 3.3. Long-Term Adverse Events

**Figure 3:**
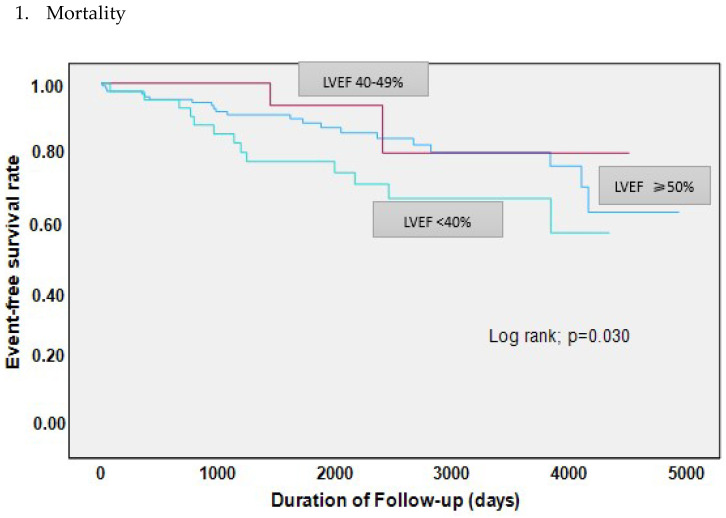

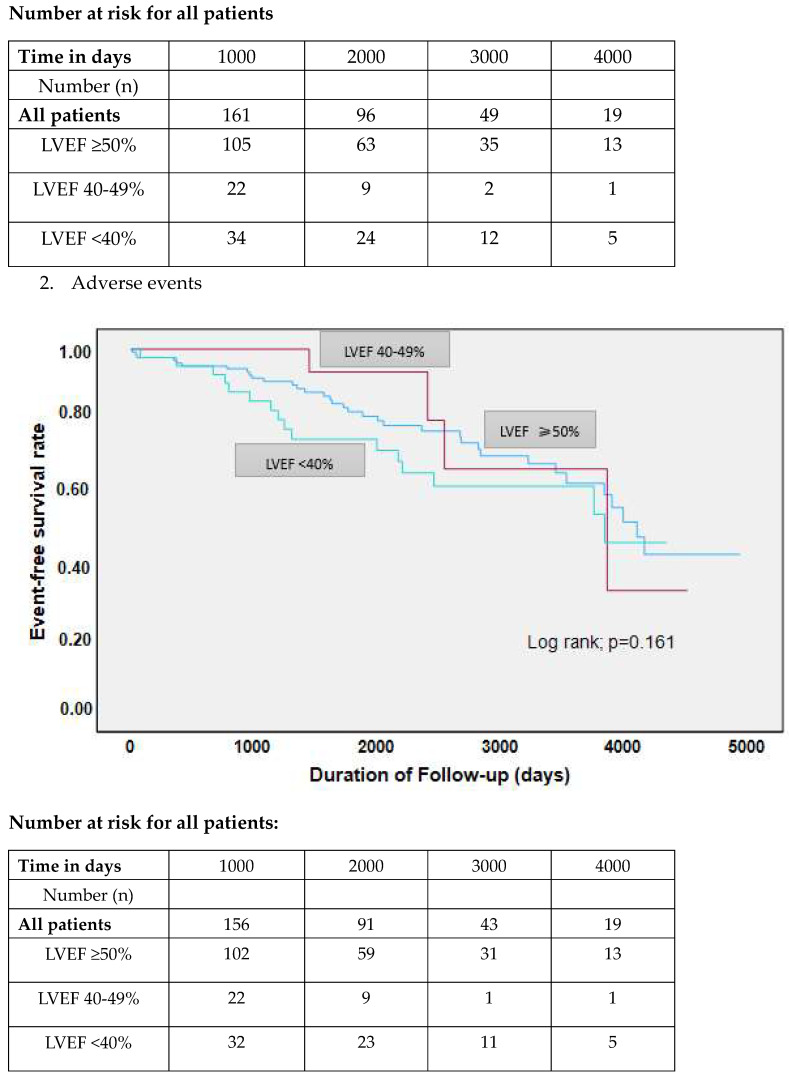
Kaplan–Meier Curves.

The composite adverse events including myocardial infarction with non-obstructive coronary artery disease and percutaneous coronary intervention were similarly presented among the groups. Nevertheless, all-cause mortality was significantly higher in the LVEF < 40% group, followed by the LVEF ≥ 50% and LVEF 40–49% groups (34.8% vs. 18.1% vs. 12.5%; *p* = 0.03). Additionally, our data showed that over a mean follow-up of 6.2 + 3.1 years, the rate of adverse events ranged between 20.8% and 41%. The distribution of adverse events was LVEF ≥ 50% (29.1%) vs. LVEF 40–49% (20.8%) vs. LVEF < 40% (41%); *p* = 0.1. Moreover, 43 patients (21%) died during the follow-up period. Of note, in 34 patients the cause of death was unknown (Table 4 and Figure 3). 

To investigate the relationships between specific co-factors and the composite in-hospital cardiovascular events, a Cox regression analysis was calculated. In the multivariable analysis, age under 50 years (HR 0.3, 95% CI: 0.1–0.8, *p* = 0.02), supraventricular tachycardia (HR = 2.1, 95% CI: 1.0–4.7, *p* = 0.04), LVEF between 49 and 40% (HR 3.0, 95% CI: 1.3–7.0, *p* = 0.008), and LVEF < 40% (HR 2.8, 95% CI: 1.4–5.6, *p* = 0.002) were predictors of the in-hospital complications (Table 5). 

## 4. Discussion

In the present study, we examined the clinical outcomes of 254 patients with suspected MINOCA diagnosis. We dissected the outcome according to different values of LVEF. The main findings are as follows: (i) baseline characteristics were different between groups and approximately half of the patients had LVEF ≥ 50%; (ii) duration of hospitalization was lowest in LVEF ≥ 50% and highest in LVEF < 40% group driven by a higher rate of in-hospital adverse events in LVEF < 40% groups compared to other groups; and (iii) over follow-up, all-cause mortality was significantly higher in the LVEF < 40% group compared to other groups.

Even though the heterogeneous profile of MINOCA is primarily associated with different mechanisms involving malfunctions in epicardial vessels, coronary microcirculation, and microvascular dysfunctions [21,22], the LVEF is associated with different outcomes in cardiovascular diseases. In the present study, we focused on the baseline characteristics and impact of different baseline LVEF values on in-hospital and long-term outcomes [15,23]. 

We included patients with a suspected MINOCA diagnosis in the present cohort. Previously, the term MINOCA was used to describe individuals with any cause of troponin elevation, including non-coronary reasons such as myocarditis or Takotsubo syndrome (TTS), as well as coronary ischemic causes. Depending on the diagnosis, the prevalence of MINOCA alone ranges from 1 to 15% [24,25]. 

In the present study, angina pectoris was the main symptom at admission in the LVEF ≥ 50% group; nevertheless, dyspnea was more commonly reported in LVEF < 40% compared to other groups [26]. Tn-I and BNP levels were significantly higher in the LVEF < 40% group compared to other groups. Increased levels of Tn-l and BNP levels are consistent with declined LVEF and a sign of higher myocardial injury [27,28]. Additionally, these parameters are related to worsened disease progression and a higher mortality rate in myocardial infarction with obstructive coronary artery disease and non-obstructive coronary artery disease [29,30]. Of note, the rate of atrial fibrillation tended to be higher in the LVEF < 40% and LVEF 40–49% groups compared to the LVEF ≥ 50% group. This may highlight the role and association of atrial fibrillation in the present cohort. Atrial fibrillation is a common relevant predictor of the outcome of patients suffering from cardiovascular disease [31,32].

Of note, malignancy and chronic kidney disease were significantly more commonly reported in the LVEF < 40% group compared to other groups. Both comorbidities could worsen the outcome of patients, which may explain the higher in-hospital complication rate and long-term mortality rate in this group compared to other groups. Subsequently, the baseline characteristics and risk profiles of patients included in different LVEF groups were variable, which may explain the variation in in-hospital outcomes. 

Heart failure is one of the common cardiovascular risk factor events in the MINOCA cohort [13]. A registry-based TOTAL-AMI study evaluated MINOCA patients at a median follow-up of 3.4 years and found that MINOCA patients had a high prevalence of heart failure and 27.6% of patients had impaired LVEF [14]. A meta-analysis reported that the prevalence of MINOCA is 6% [24,25]. Eggers et al. (2018) evaluated the data from the SWEDEHEART, including >7200 patients with MINOCA and 69,276 with first conventional AMI. MINOCA patients had the highest prevalence of heart failure and 27.6% of these patients exhibited a reduced LVEF. 

In the present study, we showed significantly higher in-hospital complications in LVEF < 40% compared to other groups. This different complication rate may be explained by the different comorbidities and cardiovascular risk factors predominated by sicker patients in LVEF < 40%. It is worth noting that, while LVEF increased in all groups at discharge, this difference did not result in complete recovery of LVEF across all groups. As a result, the duration of in-hospital stay was significantly longer in patients with LVEF < 40% compared to other groups, which may be attributed to the higher rate of adverse in-hospital events within this group. Additionally, the rates of CPR and malignant cardiac arrhythmic events were similarly high in patients with LVEF < 40% and LVEF 40–49%, as compared to those with LVEF ≥ 50%. This may support the hypothesis that heart failure with midrange EF is more similar to EF < 40% in terms of patient outcomes. It is important to note that LVEF is associated with a substantial risk of arrhythmias in myocardial infarction with obstructive coronary artery disease [13].

Previous studies have shown that MINOCA patients have fewer cardiovascular risk factors, lower rates of intra-hospital major adverse cardiovascular events (MACEs), and lower mortality rates compared to myocardial infarction with significant coronary artery disease [33,34,35]. In our study, we expanded the variability of the patient inclusion standards and shifted the prediction of the outcome of MINOCA based on LVEF. 

MINOCA is related to a higher mortality and risk profile [36]. A recent study by Armilotta et al. (2023) showed that LVEF has an impact on risk stratification in MINOCA patients [37]. Our data showed that over long-term follow-up, a significant proportion of the patients developed various adverse events. The composite adverse events, including stroke, thromboembolic events, percutaneous coronary intervention, and death, were similarly presented in the different LVEF groups. Nevertheless, all-cause mortality was significantly more present in the EF < 40% group, followed by LVEF ≥ 50% and LVEF 40–49% groups. Braga et al. (2019) conducted a comprehensive retrospective analysis to examine the prognostic significance of non-obstructive coronary disease in patients with LVEF < 40% compared to those without coronary lesions or obstructive coronary disease [38]. They reported that non-obstructive disease was independently linked to a higher risk of cardiovascular death, non-fatal AMI, non-fatal ischemic stroke, and heart failure hospitalizations. This concluded with an 18% higher rate of all-cause death compared to patients without evident coronary artery disease.

Single complications like stroke, cardiogenic shock, pulmonary edema, left ventricular thrombus, thromboembolic events, malignant arrhythmias, supraventricular arrhythmias, CPR, and malignant arrhythmias were similarly common in the different groups over follow-up. 

Of note, no research has demonstrated that a CMR diagnosis of myocarditis carries a worse prognosis compared to a normal CMR examination in patients with a working diagnosis of MINOCA. Similarly, previous studies have not demonstrated that the diagnosis of AMI is a predictor of mortality in multivariate analysis after accounting for conventional clinical indices (e.g., age, coronary artery disease [CAD] risk factors, troponin levels), despite the fact that patients with MINOCA with a CMR diagnosis of AMI appear to have moderately higher mortality than those with a normal CMR [8,39,40]. The underlying reason for MINOCA could have a significant impact on the prognosis. CMR is thought to be an important diagnostic technique for these patients. In addition, a recent study demonstrates that the presence of late-gadolinium enhancement (LGE) in CMR in MINOCA patients results in worse prognosis compared to patients not presenting LGE in CMR [41]. In our study, limited patients underwent CMR, and alternative parameters were used to define the MINOCA patients. Therefore, dividing these patients into true MINOCA vs. mimicker MINOCA was not possible, and a suspected MINOCA term was used.

Overall, it should not be neglected that MINOCA is not a benign condition but could be a result of numerous pre-existing cardiovascular risk factors and comorbidities. Our results show that LVEF differentially impacts the outcomes in this cohort and can be taken into consideration as a specific marker, and frequent follow-ups may improve the outcome of these patients. The prognosis may be ameliorated by improved care quality and closer follow-up, especially in high-risk subgroups.

## 5. Conclusions

The present study shows a significant impact of LVEF on in-hospital cardiovascular events and adverse events over long-term follow-up of MINOCA patients. Patients with LVEF < 40% and LVEF 40–49% have a similar in-hospital complication rate predominated by malignant arrhythmias, cardiopulmonary resuscitation, and respiratory support. However, the long-term adverse event rate is similar to what was reported, except for the higher all-cause mortality rate in LVEF < 40% compared to other groups.

## 6. Limitations

This investigation was carried out retrospectively in a single center over an 11-year period. The excluded patients who did not meet the criteria may have caused a selection bias. CMR was not evaluated systematically; therefore, dividing these patients into true MINOCA vs. mimicker MINOCA was not possible. Unfortunately, in the majority of patients, the cause of death could not be determined.

## Figures and Tables

**Table 1:** Baseline characteristics of 254 patients initially presenting Troponin-positive with non-obstructive coronary artery disease according to ejection fraction.

Variables	All Patients *n* = 254	LVEF ≥ 50 *n* = 170	LVEF 40–49% *n* = 31	LVEF < 40 *n* = 53	*p* Value
Age years, median (IQR)	64 (50.8–75.3)	61.5 (48–73)	67 (57–78)	68 (56–75.5)	0.051
Male—no, (%)	134 (52.1)	86 (50.6)	16 (51.6)	33 (62.2)	0.459
BMI—kg/m^2^, median (IQR)	26.6 (24–30.4)	26.8 (24.1–30.1)	25.3 (23.7–31.2)	26.9 (24.3–32)	0.870
Symptoms—no, (%)					
Angina pectoris	149 (58)	115 (67.7)	17 (54.8)	17 (32.1)	<0.001
Dyspnea	111 (43.2)	57 (33.7)	16 (51.6)	38 (71.7)	<0.001
Clinic parameter, median (IQR)					
Systolic BP, mmHg	141 (127–158)	143 (130–160)	133 (122–158)	133 (117–152)	0.039
Diastolic BP, mmHg	85 (75–96)	85 (73–94)	81 (74–95)	84 (80–95)	0.584
Heart rate, bpm, median (IQR)	88 (69–107)	82 (67–97.8)	86 (66–123)	102.5 (88.3–123)	<0.001
Medical history—no, (%)					
Current Smoking	61 (23.7)	39 (22.9)	7 (22.6)	16 (30.1)	0.716
Obesity	75 (29.2)	47 (27.7)	9 (29)	20 (37.7)	0.523
Arterial hypertension	168 (65.4)	108 (63.5)	23 (74.2)	38 (71.6)	0.423
Dyslipidemia	63 (24.5)	50 (29.4)	5 (16.1)	9 (17)	0.053
Diabetes Mellitus	41 (16)	25 (14.7)	8 (25.8)	8 (15.1)	0.298
COPD	31 (12.1)	18 (10.6)	4 (12.9)	9 (17.3)	0.462
Bronchial Asthma	21 (8.2)	15 (8.8)	3 (9.7)	4 (.5)	0.734
Malignancy	29 (11.3)	16 (9.4)	2 (6.5)	12 (22.6)	0.043
Kidney disease	32 (12.5)	14 (8.2)	5 (16.1)	14 (26.4)	0.006
Neurological disease	53 (20.6)	34 (20)	10 (32.3)	10 (18.8)	0.225
Autoimmune disease	16 (6.2)	10 (5.9)	3 (9.7)	4 (7.5)	0.712
Psychiatric disease	29 (11.3)	16 (9.4)	3 (9.7)	11 (20.7)	0.160
Pacemaker	7 (2.8)	2 (1.2)	1 (3.2)	5 (9.4)	0.044
Atrial fibrillation	57 (15.4)	20 (11.8)	8 (25.8)	12 (22.6)	0.122
Laboratory values, median (IQR)					
Troponin (µg/L)	1.8 (0.6–3.5)	1.1 (0.5–2.1)	1.3 (0.8–2.3)	3.8 (1.7–4.6)	0.018
Creatin Phosphatkinase (U/L)	150.5 (86.3–256.8)	156 (89.5–256)	127 (73–256)	133.5 (68–305.5)	0.013
BNP (pg/mL)	218 (61.5–644)	98 (48–278)	266.5 (162.5–602.3)	793 (238.3–2247.5)	<0.001
Creatinine (µmol/L)	88 (70.4–105.6)	79.2 (70.4–96.8)	88 (79–110)	105.6 (79.2–123.2)	0.003
Drugs on admission, *n* (%)					
ß-Blocker	82 (31.9)	53 (31.6)	11 (36.7)	18 (34.6)	0.885
ACE inhibitor	68 (26.5)	43 (25.6)	5 (16.7)	20 (37.7)	0.065
Statin	52 (20.2)	32 (19.1)	7 (22.6)	13 (24.5)	0.666
Sartane	36 (14)	24 (14.3)	5 (16.7)	8 (15)	0.935
Ca-Blocker	51 (19.8)	33 (19.6)	4 (13.3)	15 (28.3)	0.316
Diuretics	60 (23.3)	29 (17.3)	12 (40)	19 (35.8)	0.002
Anticoagulants	37 (14.4)	21 (12.5)	7 (23.3)	9 (17)	0.303
Aspirin	47 (18.3)	33 (19.6)	7 (23.3)	8 (15)	0.485
Clopidogrel	10 (3.9)	8 (4.8)	0 (0)	3 (5.6)	0.461
Prasugrel	1 (0.4)	0 (0)	0 (0)	1 (1.9)	0.153
Dual anti-platelet	5 (1.2)	4 (2.4)	0 (0)	1 (1.9)	0.684
Antiarrhythmics	3 (1.2)	3 (1.8)	0 (0)	0 (0)	0.525

*p*-values for the comparison between groups of BMI; BMI, body mass index; BP, blood pressure; COPD, Chronic obstructive pulmonary disease; BNP, brain natriuretic Peptide; LVEF, Ejection fraction; LVEF ≥ 50, LVEF 40–49%, and LVEF < 40. ACE, Angiotensin-converting-enzyme.

**Table 2:** Medication at discharge.

Variables	All Patients *n* = 254	LVEF ≥ 50 *n* = 170	LVEF 40–49% *n* = 31	LVEF < 40 *n* = 53	*p* Value
ß-Blocker	197 (76.7)	128 (75.3)	25 (80.7)	44 (83)	0.457
ACE inhibitor	157 (61.1)	99 (58.2)	20 (64.5)	38 (73.1)	0.202
Statin	109 (42.4)	79 (57.1)	15 (48.4)	15 (28.3)	0.053
Sartane	40 (15.6)	23 (13.5)	5 (16.1)	13 (24.5)	0.284
Ca Blocker	72 (28)	52 (30.6)	7 (22.6)	13 (25)	0.523
Diuretics	112 (43.6)	49 (28.8)	21 (67.7)	42 (80.8)	<0.001
Anticoagulants	109 (42.4)	79 (46.5)	15 (48.4)	15 (28.3)	0.053
Aspirin	10 (3.9)	5 (2.9)	1 (3.2)	4 (7.6)	0.317
Clopidogrel	112 (43.6)	76 (44.7)	15 (48.4)	21 (39.6)	0.712
Prasugrel	45 (17.7)	34 (20)	3 (9.7)	8 (15.1)	0.330
Dual anti-platelet	39 (9.6)	30 (17.7)	3 (9.7)	6 (11.3)	0.349
Antiarrhythmics	19 (7.5)	12 (7.1)	3 (9.7)	4 (7.7)	0.639

**Table 3:** In-hospital complications according to ejection fraction.

	All Patients *n* = 254	LVEF ≥ 50% *n* = 170	LVEF 40–49% *n* = 31	LVEF < 40% *n* = 53	*p* Value
Adverse event	93 (36.2)	46 (27.1)	17 (54.8)	30 (56.6)	<0.001
CPR	18 (7.1)	7 (4.1)	4 (12.9)	7 (13.2)	0.032
Left ventricular thrombus	2 (0.8)	1 (0.6)	0 (0)	1 (1.9)	0.565
Thromboembolic event	2 (0.8)	1 (0.6)	0 (0)	1 (1.9)	0.565
Pulmonary edema	5 (1.9)	3 (1.8)	1 (3.2)	1 (1.9)	0.865
Cardiogenic shock	5 (1.9)	3 (1.8)	1 (3.2)	1 (1.9)	0.865
Invasive ventilation	19 (7.4)	10 (5.9)	3 (9.7)	6 (11.5)	0.383
Non-invasive ventilation	9 (3.5)	6 (3.6)	0 (0)	3 (5.7)	0.413
Stroke	0 (0)	0 (0)	0 (0)	0 (0)	0
Duration of hospitalization—days, mean + SD	10 + 8.5	9 + 5	14 + 12	15 + 10	<0.001
Malignant cardiac Arrhythmias (on admission/in hospital)	20 (7.8)	9 (5.3)	5 (16.1)	6 (11.3)	0.068
Bradycardiac arrhythmias	5 (1.9)	1 (0.6)	1 (3.2)	3 (5.7)	0.059
- AV block 2 Mobitz	1 (0.4)	1 (0.6)	0 (0)	0 (0)	0.782
- AV block 3	1 (0.4)	1 (0.6)	0 (0)	0 (0)	0.782
- Asystole	5 (1.9)	0 (0)	2 (6.5)	3 (5.7)	0.005
Ventricular arrhythmias	12 (4.7)	5 (2.9)	4 (12.9)	3 (5.8)	0.052
- sustained	9 (3.5)	3 (1.8)	3 (9.7)	3 (5.7)	0.058
- non-sustained	10 (3.9)	2 (1.2)	5 (16.1)	3 (5.7)	<0.001
- ventricular fibrillation	7 (2.7)	4 (2.4)	1 (3.2)	2 (3.8)	0.848
Torsades de pointes	14 (5.4)	7 (4.1)	1 (3.5)	6 (11.5)	0.113
Supraventricular arrhythmias	58 (22.6)	30 (17.7)	10 (32.3)	18 (34)	0.019
Atrial fibrillation	51 (19.8)	27 (15.9)	9 (29)	15 (28.3)	0.059
- first appearance	30 (11.7)	19 (11.2)	4 (12.9)	7 (13.2)	0.906
- recurrence	17 (6.6)	7 (4.1)	5 (16.1)	5 (9.4)	0.032
Atrial flutter	5 (1.9)	2 (1.2)	1 (3.2)	2 (3.9)	0.430
- first appearance	6 (2.3)	2 (1.2)	1 (3.2)	3 (5.8)	0.180
- recurrence	1 (0.4)	1 (0.6)	0 (0)	0 (0)	0.750
In-hospital death	4 (1.6)	1 (0.6)	1 (3.2)	2 (3.9)	0.197
Cardiac caused death	3 (1.2)	1 (0.6)	1 (3.2)	1 (1.9)	0.400
Non-cardiac caused death	1 (0.4)	0 (0)	0 (0)	1 (1.9)	0.150

Adverse event, major adverse cardiac and cerebrovascular events; CPR, cardiopulmonary resuscitation; AV, atrioventricular; SD, Standard deviation; only one malignant cardiac/supraventricular arrhythmia is counted per patient (even if one patient has several arrhythmias at the same time).

**Table 4:** Extra-hospital complications (during follow up) according to ejection fraction.

	All Patients *n* = 205	LVEF ≥ 50 *n* = 134	LVEF 40–49% *n* = 25	LVEF < 40 *n* = 46	*p* Value
Adverse event	63 (30.7)	39 (29.1)	5 (20.8)	19 (41.3)	0.161
Stroke	5 (2.4)	5 (4.5)	0 (0)	0 (0)	0.264
Thromboembolic event	5 (2.4)	5 (4.2)	0 (0)	0 (0)	0.312
Recurrence of troponin-positive with non-obstructive CAD	1 (0.5)	1 (0.9)	0 (0)	0 (0)	0.794
Cardiac arrest	3 (1.5)	1 (0.9)	0 (0)	2 (6.1)	0.110
Percutaneous coronary intervention	9 (4.4)	6 (5.1)	2 (9.5)	1 (3.1)	0.594
Death	43 (21)	24 (18.1)	3 (12.5)	16 (34.8)	0.030
- cardiac caused death	3 (1.5)	2 (1.7)	0 (0)	1 (3.1)	0.703
- non-cardiac caused death	6 (2.9)	5 (4.3)	0 (0)	1 (3.1)	0.615

Adverse event, major adverse cardiac and cerebrovascular events; CAD, coronary artery disease.

**Table 5:** Multivariate analysis for the in-hospital complications.

Variable	Univariate Analysis			Multivariable Analysis		
	HR	95% CI	*p*-Value	HR	95% CI	*p*-Value
Age < 50	0.24	0.120–0.468	<0.001	0.44	0.197–0.987	0.046
Male	1.14	0.743–1.735	0.558			
Medical history						
Arterial hypertension	2.06	1.265–3.360	0.004	1.99	1.006–3.923	0.048
Diabetes Mellitus	1.19	0.686–2.069	0.535			
BMI > 30 kg/m^2^	1.01	0.972–1.050	0.608			
Supraventricular tachycardia	4.42	2.435–8.010	<0.001	2.3	0.927–5.719	0.072
Pulmonary disease	1.69	1.015–2.805	0.044	1.36	0.687–2.696	0.376
Malignancy	1.32	0.706–2.470	0.385			
Neurological disease	1.24	0.795–2.024	0.390			
Medication						
ß-Blocker	1.84	1.179–2.876	0.007	1.19	0.620–2.275	0.60
Ejection fraction						
LVEF	0.96	0.941–0.978	<0.001	0.96	0.916–1.011	0.128
LVEF 40–49%	2.75	1.279–5.914	0.010	1.48	0.486–4.514	0.489
LVEF < 40%	2.48	1.347–4.578	0.004	1.02	0.198–5.273	0.979
Laboratory values						
Troponin I	1.02	0.985–1.050	0.304			

## Data Availability

The original data presented in the study are openly available by request to the corresponding author.

## Supplementary Materials

The following supporting information can be downloaded at: https://www.mdpi.com/article/10.3390/jcm13102826/s1, Table S1: ECG parameters.
