# Supplementary material for: Ejection Fraction-Related Differences of Baseline Characteristics and Outcomes in Troponin-Positive Patients without Obstructive Coronary Artery Disease
Source: J Clin Med. 2024 May 11;13(10):2826. doi: 10.3390/jcm13102826 (PMC11121874; doi:10.3390/jcm13102826)
Supplement: Supplementary file 1 [file jcm-13-02826-s001.zip › jcm-2898009-supplementary.pdf]

Table S1. ECG parameters.

Variables	All patients n=254	LVEF≥50 n=170	LVEF 40-49% n=31	LVEF<40 n=53	P Value
**ECG — no, (%)**					
ST elevation	39 (15.2)	31 (18.3)	2 (6.5)	7 (13.2)	0.166
Inversed T-Waves	110 (42.8)	68 (40.7)	14 (46.7)	28 (53.9)	0.242
**Echocardiography data, n (%)**					
LVEF on admission (%), median (IQR)	55 (42-60)	60 (55-65)	45 (40-46)	30 (23-35)	**<0.001**
LVEF at discharge (%), median (IQR)	53 (38.3-60)	60 (56-65)	40.5 (34.8-50)	38 (27-50)	**<0.001**
Left ventricular hypertrophy	64 (24.9)	34 (20.2)	6 (20)	25 (47.1)	**<0.001**
Tricuspid valve regurgitation	69 (26.8)	39 (22.9)	9 (30)	21 (39.6)	0.055
- mild	51 (19.8)	33 (19.4)	6 (20)	12 (22.6)	0.878
- moderate	18 (7)	6 (3.5)	3 (10)	10 (18.8)	**0.003**
- severe	0 (0)	0 (0)	0 (0)	0 (0)	
Mitral valve regurgitation	82 (31.9)	46 (27.1)	11 (36.7)	26 (49)	**0.021**
- mild	64 (24.9)	39 (22.9)	8 (26.7)	18 (34)	0.406
- moderate	10 (3.9)	3 (1.8)	2 (6.7)	6 (11.3)	**0.031**
- severe	8 (3.1)	4 (2.4)	1 (3.3)	4 (.5)	0.488
Aortic valve regurgitation	35 (13.6)	24 (14.1)	5 (16.7)	7 (13.2)	0.783
- mild	29 (11.3)	20 (11.8)	4 (13.3)	6 (11.3)	0.848
- moderate	3 (1.2)	2 (1.2)	1 (3.3)	0 (0)	0.406
- severe	3 (1.2)	2 (1.2)	0 (0)	2 (3.7)	0.750

p values for the comparison between groups **LVEF**, Ejection fraction; LV**EF≥50,** LV**EF 40-49%, and LVEF<40.**
